# The L1-ORF1p coiled coil enables formation of a tightly compacted nucleic acid-bound complex that is associated with retrotransposition

**DOI:** 10.1093/nar/gkac628

**Published:** 2022-07-25

**Authors:** Ben A Cashen, M Nabuan Naufer, Michael Morse, Charles E Jones, Mark C Williams, Anthony V Furano

**Affiliations:** Northeastern University, Department of Physics, Boston, MA02115, USA; Northeastern University, Department of Physics, Boston, MA02115, USA; Northeastern University, Department of Physics, Boston, MA02115, USA; The Laboratory of Molecular and Cellular Biology, NIDDK, NIH, Bethesda, MD 20892, USA; Northeastern University, Department of Physics, Boston, MA02115, USA; The Laboratory of Molecular and Cellular Biology, NIDDK, NIH, Bethesda, MD 20892, USA

## Abstract

Long interspersed nuclear element 1 (L1) parasitized most vertebrates and constitutes ∼20% of the human genome. It encodes ORF1p and ORF2p which form an L1-ribonucleoprotein (RNP) with their encoding transcript that is copied into genomic DNA (retrotransposition). ORF1p binds single-stranded nucleic acid (ssNA) and exhibits NA chaperone activity. All vertebrate ORF1ps contain a coiled coil (CC) domain and we previously showed that a CC-retrotransposition null mutant prevented formation of stably bound ORF1p complexes on ssNA. Here, we compared CC variants using our recently improved method that measures ORF1p binding to ssDNA at different forces. Bound proteins decrease ssDNA contour length and at low force, retrotransposition-competent ORF1ps (111p and m14p) exhibit two shortening phases: the first is rapid, coincident with ORF1p binding; the second is slower, consistent with formation of tightly compacted complexes by NA-bound ORF1p. In contrast, two retrotransposition-null CC variants (151p and m15p) did not attain the second tightly compacted state. The C-terminal half of the ORF1p trimer (not the CC) contains the residues that mediate NA-binding. Our demonstrating that the CC governs the ability of NA-bound retrotransposition-competent trimers to form tightly compacted complexes reveals the biochemical phenotype of these coiled coil mutants.

## INTRODUCTION

LINE-1 (L1) is a non-LTR intragenomic DNA parasite that has been evolving in mammalian genomes for ∼100 Myr. It is the only autonomously active mobile genetic element in humans and constitutes ∼20% of human DNA (1–4). L1 can also mobilize non-autonomous transposable elements, such as Alu and SVA, and as a result L1 activity has generated upwards of ∼40% of the mass of many mammalian genomes (5–9). Despite their deleterious effects, L1 sequences remain active in most modern mammals, including humans, contributing to genetic diversity, and causing genetic defects and rearrangements. Additionally, L1 is subject to strong negative selection (10) and is a target of numerous host repressive mechanisms arrayed against other foreign genomic elements (11), indicating that it generally provides little benefit to its host. The persistence of L1 activity and its evolutionary history in mammals can, in part, be understood as an ongoing arms race (12).

**Figure 1.**
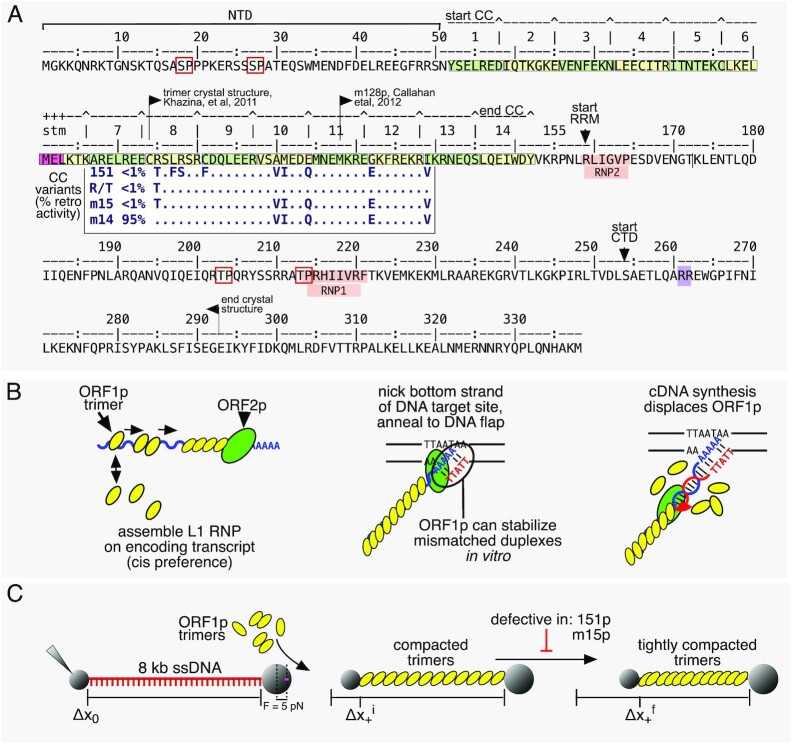
ORF1p. (**A**) Annotated sequence of ORF1p showing conserved phosphorylation sites (red boxes), the 14 heptads of the CC (alternating green and yellow boxes with a stammer (stm) in heptad 6), the highly conserved non-canonical RNA recognition motif (RRM), and C-terminal domain (CTD) that contains sequences (notably R261, R262) involved in NA-binding and chaperone activity. The N terminal domain (NTD) and terminal 46 amino acids of the CTD are intrinsically disordered (see text). The insert shows the relevant part of the alignment of the CC variants and their % retro(transposition) activity relative to the 111 (L1Pa1) wild type protein (adapted from Figure 1 in ref. 12). The amino acids that differentiate the coiled coil variants from 111p are their ancestral counterparts in the resuscitated L1Pa5 family (32). (**B**) Depiction of L1RNP assembly, involvement in, and fate during retrotransposition. (**C**) Depiction of an ssNA tethered between two beads and its length Δ*x*_0_, before and after its initial Δ*x*_+_^i^ and final Δ*x*_+_^f^ compaction.

A full-length human L1 (∼6 knt) contains a regulatory 5′ untranslated region (UTR), two open reading frames (ORFs) that encode proteins required for retrotransposition (ORF1p, ORF2p) (13,14) and a 3′ UTR which contains a highly conserved G-rich quadraplex-forming motif that stimulates retrotransposition (15,16). ORF1p and ORF2p bind their encoding transcript (*cis* preference) to form the L1 RNP, which mediates retrotransposition (17–20). ORF2p functions as the L1 replicase. It contains highly conserved endonuclease and reverse transcriptase domains that respectively nick host DNA, allowing a flap of the nicked strand to hybridize with the A-rich 3′ end of the L1 transcript, and prime its reverse transcription to generate a DNA copy that is subsequently inserted into the genome – referred to as target site primed reverse transcription (TPRT, Figure 1B) (14,21–23).

ORF1p, the major component of the L1 RNP, binds NA non-specifically with high affinity and functions as a NA chaperone, *i.e*. facilitates annealing and exchange of NA strands. It contains a 51 amino acid intrinsically disordered N-terminal domain (NTD), which harbors two highly conserved phosphorylation sites necessary for retrotransposition (24,25), followed by a 14-heptad coiled coil (26–30), which mediates trimerization of ORF1p monomers (Figure 1A). A coiled coil domain is an unusual feature of an NA chaperone and while present in ORF1 of all vertebrate L1 elements (29), and in most mammals, including humans, it is nonetheless evolutionarily labile – subject to episodic sequence changes (12). In addition, mutational analysis has shown that ORF1p activity can be quite sensitive to coiled coil substitutions (31–33).

The evolutionary lability of the coiled coil contrasts the highly conserved carboxy-terminal half of the molecule, which consists of two domains: a non-canonical RNA recognition motif (RRM) (34) that also contains two essential highly conserved phosphorylation sites, separated by an intervening intrinsically disordered loop (24,25). The RRM is followed by the carboxy-terminal domain (CTD) and the protein terminates in a 46 amino acid intrinsically disordered sequence. Several residues (*e.g*. R261 and R262) in the carboxy terminal half have been mapped to high affinity NA binding and chaperone activity *in vitro*. These activities are only evident in the context of the trimer and their mechanistic relationship to retrotransposition is not known (13,14,18,26–28,34–40).

Studies on the interaction between purified ORF1p and NA have revealed functionalities of the protein that partially recapitulate features expected of the L1 RNP, related to both its formation and its function in TPRT (28,32,41,42). For example, as illustrated in Figure 1B, generation of a productive cDNA primer for TPRT requires formation of a stable hybrid between the DNA flap and 3′ end of the L1 transcript, and ORF1p can stabilize mismatched oligonucleotide duplexes (32). Mismatches between the target site DNA primer and the A-rich 3′ terminus of the L1 transcript are likely given the degenerate nature of both the target site sequence and the 3′ terminus of the A-rich L1 transcript. In addition, the protein binds mismatched duplexes with the same affinity as it does single stranded oligonucleotides, which is 10-fold higher than to perfectly matched double stranded duplexes (28,32). These studies also showed that in addition to mediating trimerization, the coiled coil ensures the trimer-trimer interactions between nucleic acid-bound ORF1ps that support retrotransposition (Figure 1C).

Protein cross linking studies revealed that trimer-trimer interactions between NA-bound trimers are mediated by residues in the C-terminal half of the protein (28) (also see Discussion and (30) with respect to the involvement of the coiled coil). Callahan *et al.* also showed that trimer-trimer interactions enhance NA binding to oligonucleotides as those long enough to accommodate 2–3 trimers out compete binding by oligonucleotides that can accommodate only a single trimer (28). We had extended these observations using single molecule studies with force-melted ∼50 kb λ phage DNA as a source of ssNA. After initial binding, ORF1p oligomerized to a far more stably bound form (32). Reducing the force on the unwound DNA allowed double helix formation, which displaced the oligomerized ORF1p, recapitulating the dissolution of the L1 RNP during TPRT (Figure 1B). Most importantly, these studies revealed the basis of the defect in a coiled coil mutant, 151p (insert Figure 1A), which is inactive in retrotransposition (retro^–^) but biochemically the same as its retrotransposition competent (retro^+^) counterpart ORF1p (111p) for oligonucleotide-based NA binding and chaperone activity (32). The 151p protein is unable to oligomerize to a stably bound form after it initially binds to ssNA.

Our previous single molecule method for measuring ORF1p-ssDNA interactions required force-melting of dsDNA to produce local regions of effectively single-stranded DNA. Additionally, because the degree of protein binding was measured by stretching the DNA substrate periodically at discrete times, we were unable to measure the dynamics of the protein-DNA complex continuously. Here we advanced our analyses in two ways: First, we employed a recently improved single molecule method that generates ssDNA *in situ* prior to protein binding (43–45). As such, we were not limited to forces ≥60 pN that are required to unwind dsDNA, allowing us to measure ORF1p-ssNA interactions at various (lower) tensions and in real time. Second, we compared the previously studied 111p/151p pair to a new pair of retro^+^/retro^–^ coiled coil variants, m14p/m15p, which differ by a single CC residue (insert Figure 1A). These proteins are also indistinguishable in a FRET-based NA chaperone assay (Supplementary Figure S1). Unlike its active m14p counterpart, m15p, cannot form a stable, tightly compacted NA-ORF1p structure *in vitro* (Figure 1C), providing another example of the biochemical phenotype of a retrotransposition-null CC mutant. Yet, seemingly paradoxically, the CC has undergone repeated evolutionary change. However, recent analysis suggested that such evolutionary lability can protect CC function from disabling mutations (12).

## MATERIALS AND METHODS

### Purification of ORF1p

N-terminal his-tagged ORF1ps were expressed in insect cells and purified as previously described (28). This procedure produces highly purified nuclease-free phosphorylated proteins that exhibit high affinity NA binding and NA chaperone activity as determined by a FRET-based NA-annealing and strand exchange assay (24,28,32) and Supplementary Figures S1 and S3. The ORF1 sequence was derived from L1.3 a member of the L1Pa1 (L1Hs) family (NCBI L19088).

### Optical tweezers system for measuring ssDNA conformation

An 8.1 knt ssDNA molecule tethered between two functionalized microspheres (anti-DIG and Streptavidin, Figure 1C) was generated *in situ* by T7 exonucleolytic digestion as described previously (43–45) and held at various fixed tensions. Extension of the ssDNA was continuously altered to maintain the given force applied by the trapping laser in a binding buffer containing 50 mM Na^+^, 10 mM HEPES at pH 7.5. Although ORF1p trimers in the absence of NA form precipitable aggregates in 50 mM NaCl (optimal for NA binding), this does not preclude their binding to NA, or their ability to freely exchange with NA-bound trimers. However, NA-free ORF1p trimers are soluble in 0.5 M NaCl containing storage buffer (28). Therefore, concentrations of ORF1p in storage buffer were appropriately diluted into binding buffer to produce a trimer concentration of 30 nM immediately before adding to DNA. Following incubation for the indicated times, we measured the dissociation of ORF1p by replacing the protein-containing buffer with protein-free buffer. The extension of the ssDNA was controlled by a piezoelectric translational stage with 1 nm resolution, and the tension along the substrate was measured by laser deflection of the stationary optical trap (Figure 1C). Additionally, distance between the microspheres was measured using simultaneously recorded bright-field images to calculate the absolute ssDNA extension and correct for long-term thermal drift in the system. All data were analyzed using custom scripts in MATLAB (Mathworks) with uncertainty calculated as standard error of the mean of three or more replicates.

### ORF1p compaction at constant extension

The ssDNA was first incubated and fully saturated with 30 nM ORF1p trimer at a tension of 30 pN. Upon reaching equilibrium, protein-containing buffer was replaced with protein-free buffer. The extension of the protein-DNA complex was subsequently lowered to, and held at ∼0.2 nm/nt for 2, 5, 15 or 30 min. At this fixed extension, the force on the DNA varied between ∼2–10 pN depending on the incubation time (*i.e*. the tension increased with incubation time). The complex was then stretched at a rate of ∼450 nm/s until reaching 75 pN, where it was held and monitored for 100 s while it elongated. Finally, the tension was released by reducing its extension to the initial value. The stretch and release extension values at 30 pN were normalized with respect to the extension of a bare ssDNA molecule. Uncertainties were determined by the standard error of the mean of three or more replicate curves.

## RESULTS

### Binding of ORF1p to ssDNA at low force

**Figure 2.**
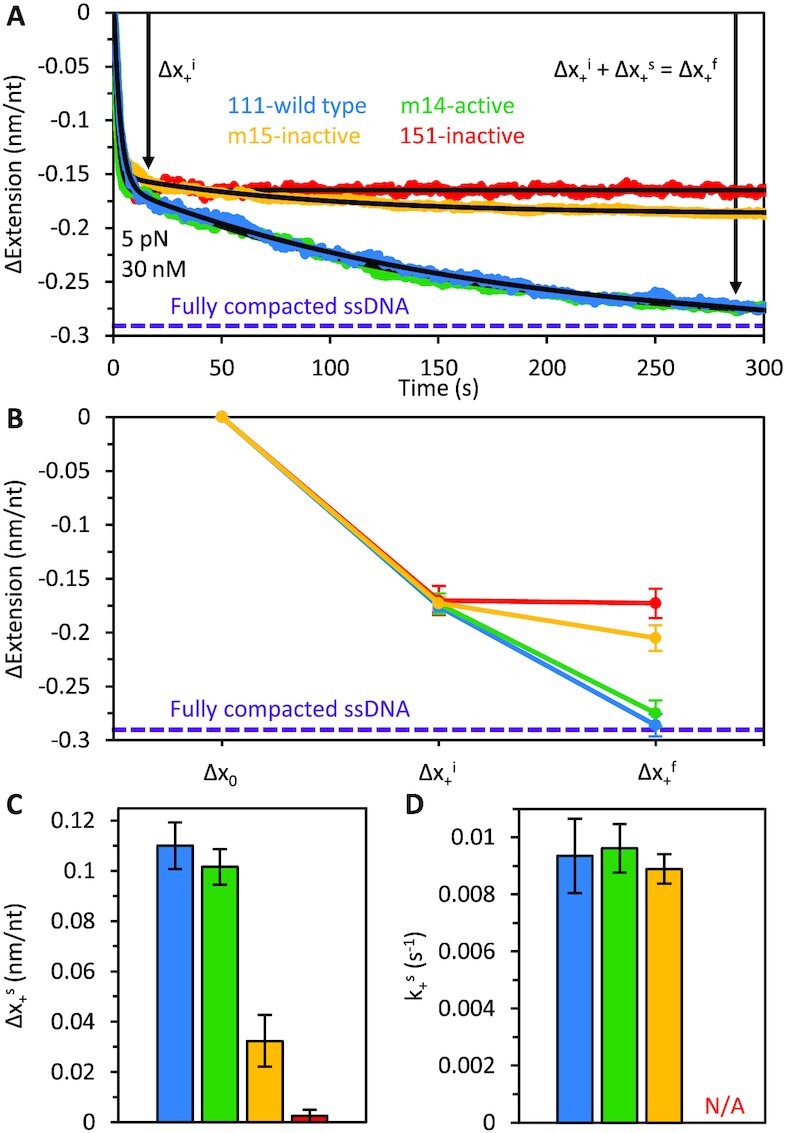
Binding of wild type and ORF1p variants to ssDNA at 5 pN. (**A**) When ssDNA is incubated with ORF1p at low force (5 pN), two phases of ssDNA compaction are observed: initial, rapid compaction (Δ*x*_+_^i^), followed by a slow, secondary compaction step (Δ*x*_+_^s^). The curves were fit with a two-rate decaying exponential function to extract a rate and amplitude associated with both phases of ssDNA compaction. The absolute extension of bare ssDNA at 5 pN is ∼0.29 nm/nt. The total ssDNA extension changes seen for 111p and m14 asymptote to ∼-0.29 nm/nt, indicating that the DNA is almost fully compacted (purple dashed line) to near zero extension. (**B**) The four proteins exhibit similar initial compaction, but the magnitude of the secondary compaction is significantly reduced for complexes formed with the inactive variants. (**C**) The amplitudes of the secondary compaction events (Δ*x*_+_^s^) are plotted as bar graphs for comparison. (**D**) The rates of secondary compaction (*k*_+_^s^) are similar for 111p, m14 and m15, however, we were unable to calculate *k*_+_^s^ for 151p as secondary compaction was negligible.

The force (tension) applied to ssDNA can affect such features as its structure (*e.g*. base stacking) and its ability to engage (conform to) protein NA-binding sites. Therefore, we determined binding of 30 nM ORF1p trimer to ssDNA at both low force (5 pN) where the ssDNA adopts a winding/flexible conformation, and high force (30 pN) where the ssDNA is effectively straightened. Figure 1C shows a schematic of the binding of ORF1p to an 8.1 knt ssDNA molecule. The data collected at 5 pN is shown in Figure 2 using the ORF1p variants listed in Figure 1A (insert – the reference sequence is L1Pa1-ORF1p, designated 111p in the text, also see Materials and Methods). Upon binding, all the variants produce an initial, rapid (τ ∼ 1 s) contraction of the DNA, Δ*x*_+_^i^_,_ but retro^+^ ORF1p variants (111p, m14p) then undergo a slower (τ ∼ 100 s), secondary compaction, Δ*x*_+_^s^, which is minimal for the retro^–^ 151p and m15p proteins. We had previously shown that unlike 111p, 151p trimers cannot form stably bound, compact oligomers on ssDNA (32). Therefore, we hypothesized that m15p, which differs from m14p by a single CC mutation (R105T), and which also produces only minor secondary compaction (Figure 2A–C), is unable to form tightly compacted structures on ssDNA. Figure 1C shows a schematic interpretation of these data.

It is important to note that unlike 151p, which contains 4 ancestral (L1Pa5) residues in heptads 8 and 9, m15p contains only 1, R105T. Due to strong epistatic (*i.e*. context dependent) effects of coiled coil mutations (12) it is not possible to predict or extrapolate the effect of a given coiled coil substitution on ORF1p activity to another context. Thus, m15p cannot be considered a subset of m151p but rather a distinct non-overlapping mutational state. On the other hand, the ORF1p coiled coil can be indifferent to multiple substitutions (12), *e.g*. the ancestral L1pa5 residues, VIQEV in heptads 10–12, do not affect ORF1p activity.

Protein binding and subsequent oligomerization on ssDNA can be modeled by the following reaction:

(1) $$\begin{equation*}{{\rm{\Theta }}}_{\rm{0}}\,\,\raise2pt{\rm \displaystyle\mathop{-\!\!\!-\!\!\!-\!\!\!\longrightarrow}^{{\rm ck}_{\rm b}}}\kern-35pt\raise-2pt{\rm \displaystyle\mathop{\longleftarrow\!\!\!-\!\!\!-\!\!\!-}_{{\rm k}_{\rm -b}}}\,\,\,\,\,{{\rm{\Theta }}}_{\rm{b}}\,\,\,\raise2pt{\rm \displaystyle\mathop{-\!\!\!-\!\!\!-\!\!\!\longrightarrow}^{{\rm k}_{\rm oligo}}}\kern-35pt\raise-2pt{\rm \displaystyle\mathop{\longleftarrow\!\!\!-\!\!\!-\!\!\!-}_{{\rm k}_{\rm -oligo}}}\,\,\,\,\,{{\rm{\Theta }}}_{\rm oligo}\end{equation*}$$

where Θ_0_, Θ_b_ and Θ_oligo_ are the ssDNA fractions of protein in the unbound, bound (but not oligomerized), and oligomerized (tightly compacted) states, respectively. *ck*_b_, *k*_−b_, *k*_oligo_, and *k_−_*_oligo_ represent the characteristic transition rates between each state. In general, the resulting differential equations are analytically intractable. However, in the case of ORF1p, the following conditions allow for an approximate analytical solution to the system of equations

(2) $$\begin{equation*}{{c}}{{{k}}}_{\rm{b}} \gg {{{k}}}_{ - {\rm{b}}}\end{equation*}$$

(3) $$\begin{equation*}{{c}}{{{k}}}_{\rm{b}} \gg {{{k}}}_{{\rm{oligo}}} + {{{k}}}_{ - {\rm{oligo}}}\end{equation*}$$

Our data supports both conditions, as ORF1p shows high binding affinity and dissociates from the NA substrate slowly (2) and reorganization of bound protein occurs over a longer timescale than its initial binding (3). These conditions indicate that the bound state, Θ_b_, effectively reaches full occupancy, and that bound ORF1p saturates the substrate on a much shorter timescale than subsequent oligomerization, allowing us to decouple the transitions between each state (*i.e*. the transitions occur sequentially). This gives the following solution:

(4) $$\begin{equation*}\Delta {x} ({t}) =\Delta {x}_{+}^{\rm i}\left(1- {\rm e}^{-k_{+}^{\rm i}t}\right)+\Delta {x}_{+}^{\rm s}\left(1- {\rm e}^{-k_{+}^{\rm s}t}\right)\end{equation*}$$

where Δ*x*_+_^i^ and *k*_+_^i^ = *ck*_b_ are the respective magnitude and rate of initial compaction due to ORF1p binding. Δ*x*_+_^s^ = α(*n* – 1)*k*_oligo_/(*k*_oligo_ + *k*_−oligo_) where α is proportional to the magnitude of compaction per protein oligomerization event, *n* represents the number of proteins bound to the ssDNA, and *k*_+_^s^ = *k*_oligo_ + *k*_−oligo_.

The rates (*k*_+_^i^) and amplitudes (Δ*x*_+_^i^) of initial compaction are equivalent for the four trimers, indicating that they initially bind the substrate identically at 5 pN (Supplementary Table S1). However, the magnitude of the oligomerization-driven, secondary compaction, Δ*x*_+_^s^, is significantly reduced for the inactive variants (Figure 2A–C). As we have no information on the oligomerization on and off rates, *k*_oligo_ and *k*_−oligo_, we can only measure the sum of the two rates, *k*_+_^s^ (which is similar for 111p, m14p and m15p, Figure 2D). Possible explanations for the observed differences in compaction include: (i) The degree of compaction from each oligomerization event, α, differs between the active and inactive proteins but the rates, *k*_oligo_ and *k*_−oligo_, are unchanged. (ii) α is unchanged, but *k*_oligo_ and *k*_−oligo_ change such that their sum remains the same, and transition into the oligomeric state, Θ_oligo_, is inhibited (*i.e*. the fraction of protein in the oligomeric state at equilibrium, *k*_oligo_/(*k*_oligo_ + *k*_−oligo_), decreases). (iii) These quantities, α, *k*_oligo_ and *k*_−oligo_ are all changed. While the cause of the reduction in ORF1p-ORF1p compaction is not known, its ability to do so and thereby support retrotransposition is exquisitely sensitive to the CC sequence (12).

### Assessing inter-trimer interactions that develop at low force

**Figure 3.**
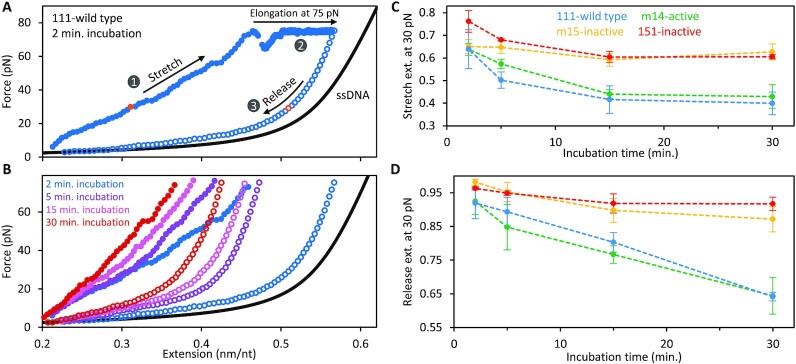
Dynamics of ORF1p-ssDNA compaction at low, fixed extension. (**A**) ssDNA previously incubated for 100 seconds at 30 pN with 30 nM 111p was held at a minimal, fixed extension (∼0.2 nm/nt) in protein-free buffer for 2 minutes. The protein–ssDNA complex was then stretched until reaching a tension of 75 pN (1), where it was held for 100 s (2). Tension on the strand was then released by reducing its extension to the initial value (3). (**B**) Incubation of the complexes formed by 111p in protein-free buffer at low extension (0.2 nm/nt) was repeated for 5, 15 or 30 min. The ssDNA extension for both the initial stretch (closed circles) and subsequent release (open circles) is inversely proportional to the initial incubation time. (**C**) Extension of the pre-formed ssDNA–ORF1p complex at 30 pN during stretching (normalized to the length of protein-free ssDNA) shows that the compaction of the active protein–DNA complexes (111p and m14) is greater than that of the inactive proteins (151p and m15). (**D**) Similarly, during release, the reduction of extension at 30 pN is greater for the active proteins than the inactive variants.

We examined the properties of ORF1p–ssDNA complexes that form at low force by subjecting them to cycles of extension and release after first incubating them under protein-free conditions for 2, 5, 15 and 30 min at a low fixed extension (∼0.2 nm/nt, Figure 3, see Materials and Methods). After 2 min, the resulting stretch curve resembles that of a polymer with significantly reduced contour length relative to bare ssDNA (Figure 3A, B). The average slope of the stretch curve of the protein-DNA complex increases with the duration of initial low force incubation, consistent with their reduced elasticity, likely an effect of increasing compaction due to interactions between NA-bound trimers. Additionally, following 100 s at 75 pN the release curves reveal shortening of the ORF1p–ssDNA complex concomitant with the time of the initial low force incubation. This indicates that ORF1p-ssDNA complexes that form at low force can convert to higher order compacted structures in the absence of free protein. Moreover, the shift in the extension of the release curves indicate that the compacted structures become increasingly stable with time, resisting disruption at very high force. At 75 pN the extension-time profile of the complex (Supplementary Figure S2) shows a series of gradual increases in extension, presumably reflecting dissolution of the higher order compacted protein–DNA structures. The increases in extension show a high degree of variability, ranging from tens to several hundreds of nanometers, indicating that the compact structures formed at low force, although quasi-stable, may be quite large and nonuniform in size.

Figure 3C and D shows that the ORF1p-ssDNA complexes formed by retro^+^ 111p and m14p undergo significantly greater (and more stable) compaction than the retro^–^ 151p and m15p proteins. These results recapitulate the findings on the complexes that form at 5 pN (Figure 2) and together corroborate our earlier studies (32) that the ability of NA-bound ORF1p trimers to form stable higher order complexes is positively correlated with retrotransposition activity, and as we showed here both properties can be abolished by the single R105T CC substitution.

### Binding of ORF1p to ssDNA at high force

**Figure 4.**
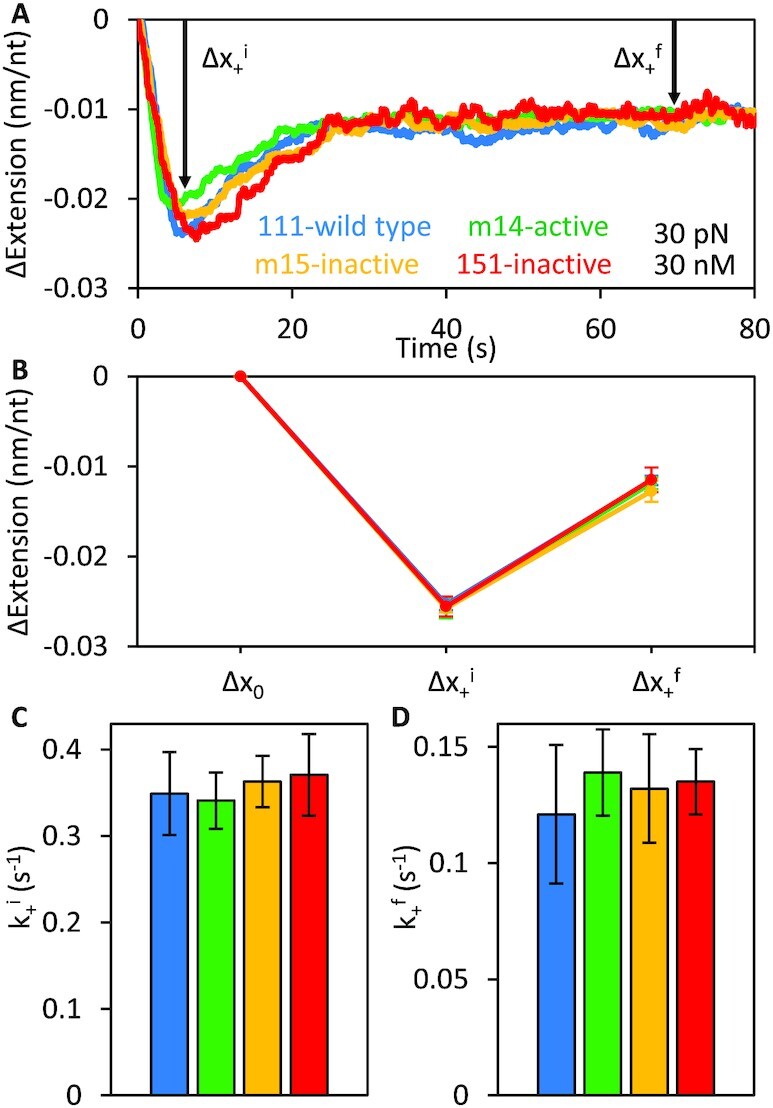
Initial binding phases of active (111p, m14) and inactive (151p, m15) trimers at 30 pN. (**A, B**) The extension changes, Δ*x*_+_^i^ and Δ*x*_+_^f^, of ssDNA (absolute extension of bare ssDNA at 30 pN is ∼0.54 nm/nt) during incubation with wild type ORF1p and the three coiled coil variants (m14, m15 and 151p) show identical biphasic binding behavior, indicating that the proteins initially bind ssDNA in a similar manner at 30 pN. The initial rates of ssDNA compaction (*k*_+_^i^), (**C**) and subsequent elongation (*k*_+_^f^), (**D**) due to ORF1p binding are equivalent for the four trimers.

The binding dynamics of the ORF1 proteins at 30 pN (Figure 4), at which the substrate tension is high enough to disfavor compaction, are dramatically different from what occurs at 5 pN (Figure 2). Regardless of their ability to support retrotransposition, the proteins induce the same initial, rapid (τ ∼ 1 s) ssDNA compaction (Δ*x*_+_^i^) followed by a slower (τ ∼ 10 s) partial elongation, equilibrating to the same final extension (Δ*x*_+_^f^) less than that of bare ssDNA (Figure 4). Similar to the behavior at low force (Figure 2), this biphasic binding signature indicates a change in the conformation of the protein-DNA complex over time. However, in contrast to what is seen at 5 pN, the oligomerization-deficient 151p binds identically to wild type, suggesting that these changes in ssDNA extension are not primarily driven by trimer-trimer interactions between NA-bound ORF1p.

**Figure 5.**
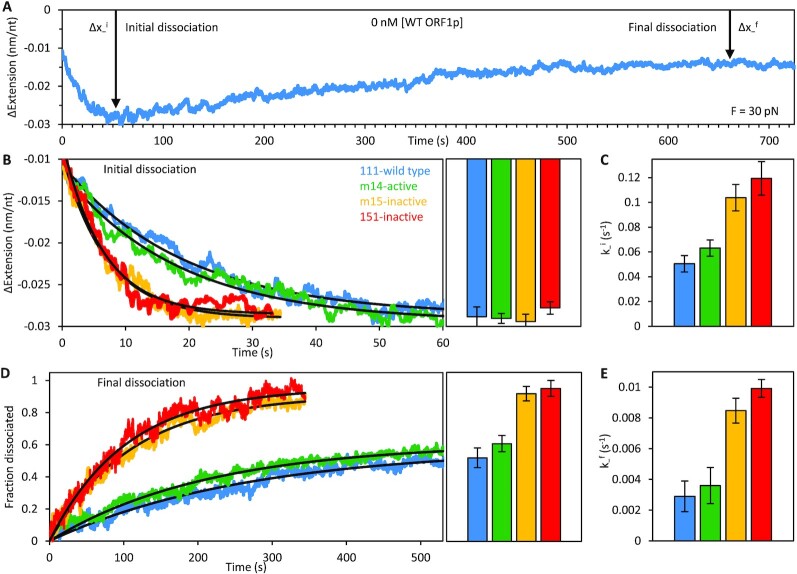
Dissociation phases of active (111p, m14) and inactive (151p, m15) trimers at 30 pN. (**A**) Representative 111p dissociation curve showing two phases of dissociation at 30 pN: an initial re-compaction (Δ*x*_−_^i^) followed by ssDNA elongation (Δ*x*_−_^f^). (**B**) All ORF1p trimers eventually compact the DNA to the same extent during the initial dissociation phase. Bar graphs show the average amplitude of compaction from multiple (*n* ≥ 3) experiments with each variant. However, re-compaction of the ssDNA occurs approximately twice as fast with the inactive variants as it does with the active proteins (**C**). Final dissociation of the inactive ORF1 proteins is both more complete (**D**) and faster (**E**) than the active trimers. In contrast, the ssDNA binding dynamics of all three ORF1p variants (m14, m15 and 151p) are identical to those of the wild type at 30 pN (shown in Figure 4).

Although the variants show similar biphasic binding at high force, when free unbound ORF1p is removed from the channel (Figure 5), differences between the retro^+^ and retro^–^ protein complexes are readily apparent: While all exhibit an initial rapid (τ ∼ 10 s) decrease in extension (Δ*x*_−_^i^), followed by a slower (τ ∼ 100 s) elongation to a final extension that does not attain that of bare ssDNA (Δ*x*_−_^f^), both the rate and magnitude of these extension changes are significantly greater for the retro^–^ 151p and m15p proteins. Specifically, re-compaction of the ssDNA during the initial dissociation phase is ∼2-fold faster for the inactive trimers. Moreover, the final dissociation of 151p and m15p is both more complete (*i.e*. the ssDNA approaches its protein-free conformation) and faster (nearly 3-fold) than the active trimers, indicating that they are less stably bound to ssDNA. This would be expected if the trimers are unable to form tightly compacted oligomers on ssDNA, supporting our conclusions from the binding experiments at low force (Figures 2 and 3) and our prior studies on 151p and 111p using our previous single molecule method (32).

## DISCUSSION

Here we extended our single molecule studies on ORF1p coiled coil variants that had shown a relation between retrotransposition and the ability of ssNA-bound ORF1p to form stably bound nucleoprotein (NP) complexes (32). With our recently improved method we re-examined this interaction by probing an additional pair of retro^+^ and retro^–^ ORF1ps. We determined the interaction of the ORF1ps as a function of force (tension) applied to the NA, a critical parameter that both governs formation and reveals properties of the ORF1p-NA complex. At 5 pN, retro^+^ 111p and m14p formed tightly compacted NPs with ssDNA that were not attainable by the retro^–^ CC mutants 151p and m15p (Figures 1C–3). These results recapitulate and extend our earlier study (32) as they include analysis of the additional pair of retro^+^ m14p and retro^–^ m15p CC variants, which differ by a single CC residue (Figure 1A). The four proteins were indistinguishable by an oligonucleotide-based chaperone (FRET) assay (Supplementary Figure S1) as we had shown earlier for retro^+^ 111p and retro^–^ 151p (32).

At 5 pN, all ORF1ps, regardless of retrotransposition competence, attained the same initial ‘compacted’ state, Δ*x*_+_^i^, and at essentially the same rate, *k*_+_^i^, (Figure 2A, Supplementary Table S1). Studies (28,32) using oligonucleotide-based binding assays also revealed no difference in binding affinity between 111p and 151p. Thus, the initial compacted state (Figure 2) might be similar to that attained by ORF1p bound to oligonucleotides (28). NA-bound trimers were close enough (∼16 Å) to be cross linked by the bifunctional cross-linking reagent EGS [ethylene glycolbis(succinimidylsuccinate)] and involved inter-trimer contacts located in the carboxy terminal half of ORF1p (28). These findings were consistent with atomic force microscopy images showing NA-bound mouse ORF1p aligned side by side on ssRNA involving contacts between the carboxy terminal half of the trimer (35).

In contrast, subsequent transition to a ‘tightly compacted’ state (Δ*x*_+_^f^), was only attained by retro^+^ 111p and m14p (Figure 2, Supplementary Table S1). At a tension of 5 pN, the absolute extension of our bare 8.1 knt ssDNA molecule was measured at ∼2.35 μm (normalized to 0.29 nm/nt), consistent with the freely jointed chain polymer model (46). The average total extension changes (Δ*x*_+_^f^) seen for the 111p and m14p complexes approached ∼−0.29 nm/nt, indicating that over long incubation timescales this tightly compacted state corresponds to a conformation wherein the DNA is almost fully compacted to near zero extension (Figure 2, purple dashed line). Furthermore, these tightly compacted structures were more resistant to disruption than the complexes formed by retro^–^ ORF1ps. Following incubation at a minimal, fixed extension (∼0.2 nm/nt), the ORF1p-NA complexes were subjected to high force (75 pN) for 100 seconds (Figure 3, Supplementary Table S2). Upon reaching 75 pN, the ssDNA elongated, likely reflecting re-organization of NA-bound ORF1p complexes. Over time, retrotransposition competent trimers remained tightly compacted but the NA complexes formed by retro^–^ proteins reverted to nearly the extension of bare ssDNA. Thus, in addition to tight compaction, retrotransposition is positively correlated with stability of the compacted ORF1p-NA complex.

Although ORF1p binding at 30 pN produces a biphasic change in extension, it differs from what occurs at 5 pN. The length reduction attained in the first phase is about 7-fold less than at 5 pN and, rather than not changing (retro^–^) or undergoing further shortening (retro^+^), the ORF1p-ssNA complex re-elongates almost 2-fold, though to less than that of bare ssDNA, indicating that ORF1p remains bound to the DNA (*cf*. Supplementary Tables S1 and S3 and Figures 2 and 4). Furthermore, in contrast to the 5 pN data, the biphasic length profiles of retro^+^ and retro^–^ ORF1p are nearly identical. These results likely reflect the different rigidities of ssNA at 30 and 5 pN, which would alter the binding options available to the protein. Additionally, Figure 5 shows that the 30 pN ORF1p–ssNA complexes formed by retro^–^ proteins are significantly more labile than those assembled by retro^+^ ORF1p, losing protein faster and more completely than the retro^+^ complexes in protein-free buffer (Figure 5, Supplementary Table S4). This likely results from the weak or even lack of inter-trimer interaction between NA-bound retro^–^ ORF1p, because the inherent affinity of retro^+^ and retro^–^ are the same for NA in both the single molecule assay (Δ*x*_+_^i^, phase 1, Figure 2, Supplementary Table S1) and oligonucleotide-based assays (28,32). Therefore, we felt justified in modelling the nature of the 5 pN complexes on our cross-linking evidence which showed trimer-trimer contacts between oligonucleotide-bound ORF1p. However, understanding the nature of the complexes that form at high force will require further quantification under different solution and substrate conditions (*e.g*. protein concentration, DNA tension, *etc*.) and, potentially, the development of additional analytical techniques. On the other hand, force-dependent generation of distinct ORF1p-ssNA complexes illustrates the advantages of our current single molecule method to reveal the different possible modes of NA–ORF1p interaction.

Our earlier studies indicated that the carboxy-terminal half of ORF1p can mediate the inter-trimer interactions responsible for oligomerization of NA-bound ORF1p (28,32). Before discussing these results in the current context, we address two topics relevant to trimer-trimer interaction. First, it was recently suggested that the coiled coils of different trimers could mediate their interaction (30). These conclusions were based on the interaction of coiled coils that had been solubilized to monomers in guanidinium HCl from inclusion bodies (insoluble aggregates) that had accumulated during their synthesis in *Escherichia coli*. Fully denatured ORF1p coiled coils such as these, which also lack the NTD, are likely an in vitro artifact that would not exist in ‘nature’. It is almost certain that ORF1p monomers trimerize while being synthesized on adjacent ribosomes (47), and we are not aware of any evidence showing that the coiled coils of fully formed trimers synthesized *in vivo* can unravel to mediate interactions between different trimers.

The second topic is ORF1p aggregation. This topic dates to 1996 (48) and was addressed again in 2012 using highly purified ORF1p to determine the effect of salt and nucleic acid on this process (28). These earlier studies employed chemical cross linking and gel electrophoresis and showed that ORF1p aggregates can form in either the presence or absence of NA. While NA-free ORF1p trimers are soluble in 0.5 M NaCl, they form precipitable aggregates in 50 mM NaCl (optimal for NA binding), which does not preclude their binding to NA, or their ability to freely exchange with NA-bound trimers (28,49). ORF1p aggregation has also been addressed in two recent reports respectively by Newton *et al.* (50) and Sil *et al.* (51). These studies used microscopically visible phase separated condensates (droplets) as a measure of ORF1p aggregation, droplet formation being likely mediated by the NTD, an intrinsically disordered region (IDR) (52). Both the Newton and Sil studies imply that condensate formation is intrinsic to the function of ORF1p in retrotransposition. However, there is no evidence for this assumption and the functional relevance of condensate formation by IDR-containing proteins in general has been questioned by Martin *et al.* (52), who stated ‘The notion that the presence of an IDR means a protein has evolved to phase separate is an inaccurate inference that has unfortunately been used to justify questionable lines of inquiry and questionable experimental design*’*. This admonition is exemplified by Newton *et al.* who showed that phase separated condensates require just the N-terminal 152 amino acids (NTD + coiled coil). As this region of ORF1p does not contain the highly conserved residues in the RRM and CTD shown by mutational analysis (18,34,39) to be in involved in NA binding and RNP formation, condensate formation is indifferent to these RNA binding domains that are critical to the role of ORF1p in retrotransposition.

How the CC ensures the formation of tightly compacted NA-bound ORF1p, and why it is required for retrotransposition remain open questions. As to the former, this role of the CC is highly sensitive to its sequence. Retro^+^ m14p and retro^–^ m15p differ by a single CC residue and we have identified a number of single CC substitutions that just as dramatically affect ORF1p activity – either abolishing it or fully restoring it (12). The phenotypic effects of CC mutations often depend on their sequence context. These are termed epistatic, and evolutionary responses to buffer negative epistatic mutations have at times governed CC evolution (12). The CC could govern the relative orientation of the carboxy-terminal half of the monomers that constitute the trimer (28). Atomic force microscopy of the mouse trimer and X-ray crystallography of the human trimer showed that this region assumes a propeller-like structure (27,35). Therefore, it is conceivable that a torque-altering coiled coil substitution could be transmitted through its length (53) and reduce the efficiency of the trimer-trimer contacts that mediate their ability to form tightly compacted NA-bound structures associated with retrotransposition.

Several possibilities could account for the correlation between retrotransposition and tightly compacted trimers. If this phenomenon also applies to the L1RNP, then in addition to protecting the parent transcript from nucleases and APOBEC3 deaminases, tight side-by-side packing of trimers could prevent formation of RNA secondary structures (35). Due to hydrogen bonds contributed by the ribose 2′0H, uncoated RNA is prone to form stable, even if short ranged, secondary structures (54,55) that could seriously impede reverse transcription. Although such possibilities seem reasonable, until we understand ‘tightly packed’ in structural terms, they remain speculative.

## DATA AVAILABILITY

The experimental data sets are either included in the main text, supplementary material, or are available from the authors upon request.

## Supplementary Material

gkac628_Supplemental_File
