# Supplementary material for: The L1-ORF1p coiled coil enables formation of a tightly compacted nucleic acid-bound complex that is associated with retrotransposition
Source: Nucleic Acids Res. 2022 Jul 25;50(15):8690–9. doi: 10.1093/nar/gkac628 (PMC9410894; doi:10.1093/nar/gkac628)
Supplement: gkac628_Supplemental_File [file gkac628_supplemental_file.pdf]

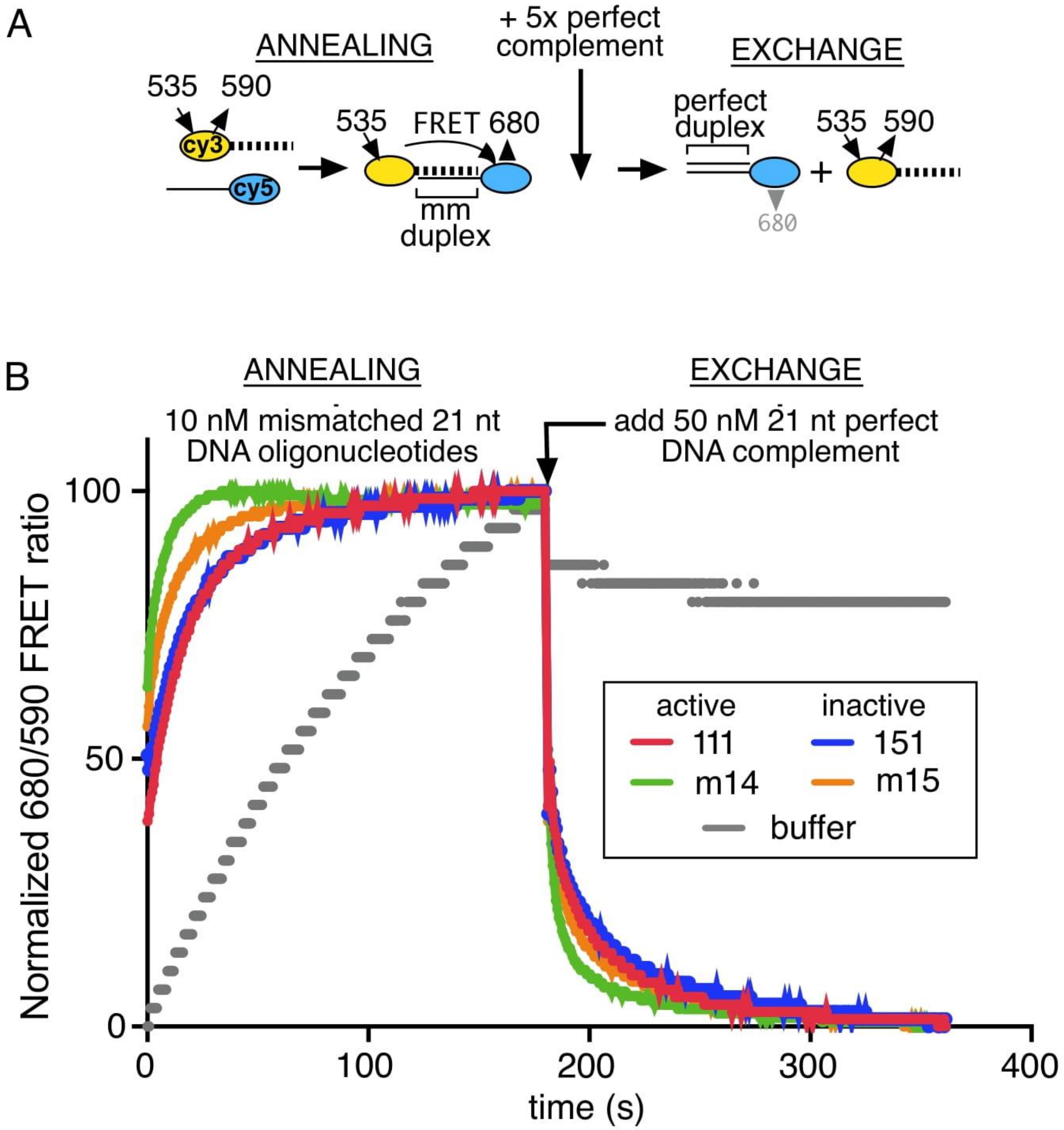

Figure 1S: FRET-based NA chaperone assay

This assay was carried out as described in the legend of Figure 3 of reference 32 of the main paper.

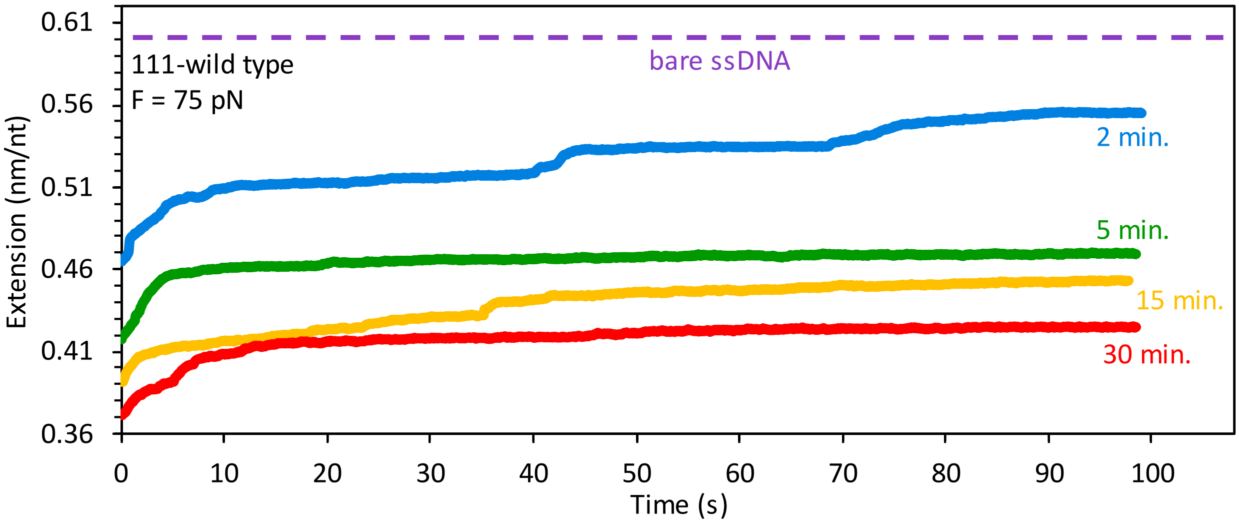

Figure 2S: ORF1p-ssDNA elongation at high force

Gradual elongation events which occur at high force (75 pN) following incubation (2, 5, 15, and 30 min.) of 111p at a minimal, fixed extension of 0.2 nm/nt (corresponding to step 2 outlined in Fig. 3). As incubation time is increased, the complex remains more compact at high force. Purple dashed line indicates extension of bare ssDNA at 75 pN for reference.

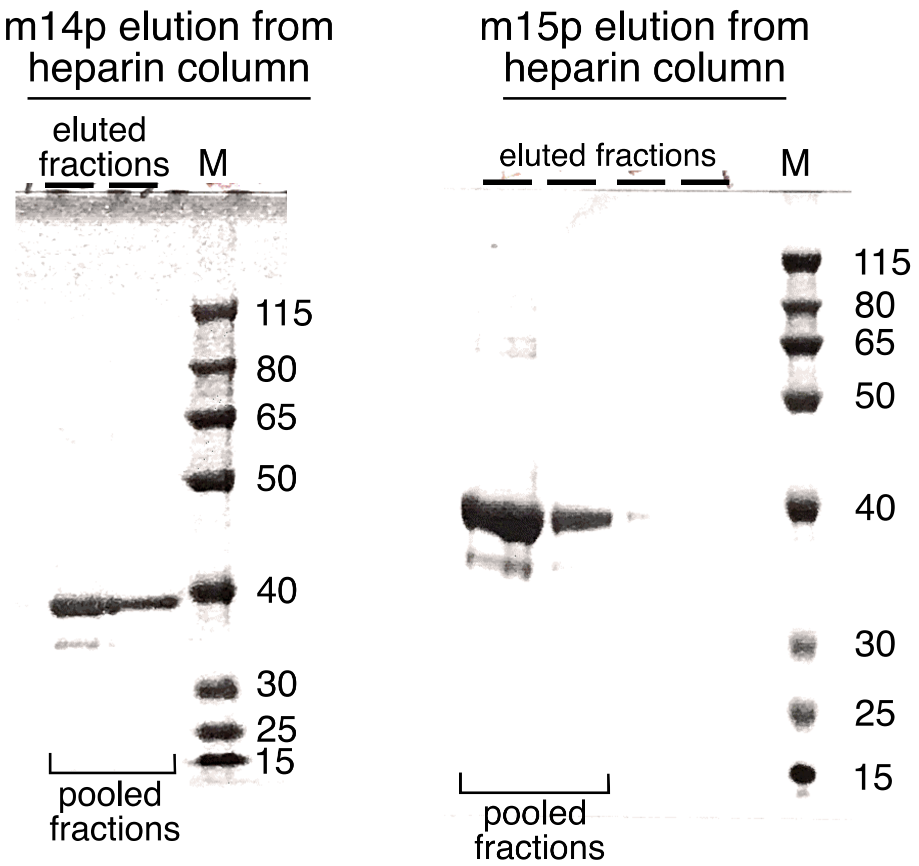

Figure 3S: Denaturing gel electrophoresis of m14p and m15p ORF1 proteins

Denaturing polyacrylamide gel electrophoresis of the final purification step of the m14p and m15p ORF1 proteins expressed and purified as described in the Materials and Methods and in full detail in (28). Electrophoresis was performed per the supplier's instructions on 10% 1mm Bis-Tris NuPAGE Minigels (Thermo Fisher – NP0301) and electrophoresed for about 1 hour at room temperature and 200 constant V using 1x NuPAGE® MOPS SDS Running Buffer (NP0001) and stained with Coomassie Blue.

Table 1S: Dynamics of ORF1p-ssDNA compaction at low, fixed force

F = 5 pN	111p	m14p	151p	m15p
Δx_+^i (nm/nt)	-0.176±0.004	-0.173±0.009	-0.170±0.014	-0.173±0.006
k_+^i (s^{-1})	0.430±0.038	0.455±0.064	0.424±0.053	0.432±0.034
Δx_+^s (nm/nt)	-0.110±0.009	-0.102±0.007	-0.002±0.002	-0.032±0.009
k_+^s (s^{-1})	0.0094±0.0013	0.0096±0.0009	N/A	0.0089±0.0005
$\Delta x_+^f = \Delta x_+^i + \Delta x_+^s$ (nm/nt)	-0.286±0.010	-0.275±0.012	-0.173±0.014	-0.205±0.011

Extension changes and rates for 111p and mutant ORF1p-DNA complexes generated with 30 nM protein at 5 pN. Data were fit to a two-decaying exponential function: $\Delta x(t) = \Delta x_+^i(1 - e^{-k_+^i t}) + \Delta x_+^s(1 - e^{-k_+^s t})$ where, respectively, Δx_+^i and k_+^i are the magnitude and rate of the initial, "fast" compaction due to ORF1p binding, and Δx_+^s and k_+^s are the magnitude and rate of the secondary, "slow" compaction due to interprotein interactions (see Fig. 2). "N/A", not calculated as compaction was negligible.

Table 2S: Dynamics of ORF1p-ssDNA compaction at low, fixed extension

x = 0.2 nm/nt	Incubation time (min.)	111p	m14p	151p	m15p
Stretch extension at 30 pN	2	0.639±0.086	0.638±0.027	0.762±0.048	0.651±0.031
	5	0.502±0.036	0.573±0.021	0.681±0.005	0.647±0.028
	15	0.416±0.061	0.440±0.019	0.604±0.025	0.592±0.029
	30	0.399±0.050	0.429±0.052	0.606±0.009	0.627±0.035
Release extension at 30 pN	2	0.919±0.046	0.925±0.040	0.963±0.005	0.981±0.009
	5	0.893±0.042	0.848±0.068	0.949±0.007	0.952±0.029
	15	0.803±0.029	0.766±0.026	0.918±0.028	0.898±0.036
	30	0.641±0.013	0.644±0.055	0.917±0.020	0.871±0.038

Normalized 30 pN extension values from ORF1p stretch and release cycles upon incubation at ~0.2 nm/nt. All values were normalized with respect to the extension of bare ssDNA and taken with an initial incubation concentration of 30 nM (see Fig. 3).

Table 3S: Binding phases (30 nM) of active (111p, m14) and inactive (151p, m15) trimers at 30 pN

F = 30 pN	111p	m14p	151p	m15p
Δx_+^i (nm/nt)	-0.025±0.001	-0.026±0.001	-0.026±0.001	-0.026±0.001
k_+^i (s^{-1})	0.349±0.048	0.341±0.032	0.371±0.047	0.363±0.030
Δx_+^f (nm/nt)	-0.012±0.001	-0.012±0.001	-0.012±0.001	-0.013±0.001
k_+^f (s^{-1})	0.121±0.030	0.139±0.019	0.135±0.014	0.132±0.023

Extension changes and rates associated with the binding phases of wild type ORF1p and the coiled coil variants. All data were taken at 30 pN with a protein incubation concentration of 30 nM (see Fig. 4).

Table 4S: Dissociation phases (in the absence of protein) of active (111p, m14) and inactive (151p, m15) trimers at 30 pN

F = 30 pN	111p	m14p	151p	m15p
Δx_{-}^{i} (nm/nt)	-0.029±0.001	-0.029±0.001	-0.028±0.001	-0.029±0.001
k_{-}^{i} (s^{-1})	0.050±0.007	0.063±0.007	0.119±0.014	0.104±0.011
Fraction diss.	0.519±0.062	0.607±0.050	0.950±0.050	0.918±0.045
k_{-}^{f} (s^{-1})	0.0029±0.0010	0.0036±0.0012	0.0099±0.0006	0.0085±0.0008

Extension changes and rates associated with the dissociation phases of wild type ORF1p and the coiled coil variants. All data were taken at 30 pN with a protein incubation concentration of 30 nM. The fraction of ORF1p that dissociated from the ssDNA was approximated as $1 - \Delta x_{-}^{f}/\Delta x_{-}^{i}$ where Δx_{-}^{f} is the final, equilibrium extension change of the protein-DNA complex in the absence of free protein and Δx_{-}^{i} is the extension change of the re-compacted complex preceding final dissociation (see Fig. 5).
